# Persistence of higher visual function deficits in adults with early-onset cerebral visual impairment: A comparative study

**DOI:** 10.1371/journal.pone.0354174

**Published:** 2026-08-21

**Authors:** Arvind Chandna, Silvia Veitzman, Celine Kim, Mike Wong, Joseph Towler

**Affiliations:** 1 Alder Hey Children’s Hospital, Liverpool, United Kingdom; 2 Smith-Kettlewell Eye Research Institute, San Francisco, California, United States of America; ISSEP Kef: Universite de Jendouba Institut Superieur du Sport et de l’Education Physique du Kef, TUNISIA

## Abstract

Cerebral Visual Impairment (CVI) of early onset has primarily been studied in children, but little is known about its progression into adulthood, despite CVI being recognized as a lifelong condition. This study investigates higher visual function deficits (HVFDs) in adults with early-onset CVI through a prospective controlled design. We first assessed the feasibility of an adapted HVF Question Inventory (HVFQI-59Q) in detecting HVFDs in adults with CVI compared to neurotypical (NT) adults (ANTs, n = 67). We then compared HVFD patterns in adults with early-onset CVI (A–Early Onset, n = 29), adults with late-onset brain injury (A–Late Onset, n = 22), and a previously interviewed cohort of children with CVI (n = 92) and child neurotypicals (n = 120). The HVFQI-59Q was used to evaluate the spectrum and severity of HVFDs across the adult groups. Results showed HVFDs are present in A–Early Onset, with similarities and distinct differences compared to children. The A–Late Onset group exhibited a broader range of deficits and lower median values, suggesting more variability in visual impairments. These findings confirm that HVFDs persist into adulthood and that late-onset brain injuries may result in HVFDs similar to CVI, challenging previous assumptions about localized effects and the need for a review of terminology for A–Late Onset, highlighting the need for further research in the adult group. The study underscores the importance of continuous CVI evaluation across the lifespan and supports the HVFQI-59Q as an effective clinical tool for detecting HVFDs.

## 1. Introduction

Cerebral visual impairment (CVI) from brain injury at or around birth (early onset) is a lifelong condition that has now been extensively studied in children [1]. Though CVI has been described as a lifelong condition [2], the profound effects of higher visual function deficits (HVFDs) in adults with early-onset CVI remains largely unexplored. Only a handful of case reports in adults with CVI describe the persistence of challenges with functional vision and have provided a glimpse into the challenging lived experiences of this population [1]. In our prior research, we examined Higher Visual Function Deficits (HVFDs) in early-onset CVI in children 4–18 years of age with normal VA [3]; with different levels of VA loss [4] and reported on lived experiences of three adults and an older child with early onset CVI [5]. In this report, we present findings from our prospective controlled study investigating the spectrum and severity of HVFDs in adults with early-onset CVI (A–Early Onset). We also compare these results with data from our earlier pediatric cohort [4] children with early-onset CVI (C–Early Onset) and with adults who sustained brain injuries in adulthood (after the age of 18) which we describe as Adults with Late Onset Brain Injury (A–Late Onset) as their challenges with brain damage have been termed distinct from early-onset CVI [6].

CVI in childhood commonly arises due to adverse events occurring during early development, particularly at or around the time of birth. It is characterized by a combination of variable visual acuity (VA) loss and higher visual function deficits (HVFDs), which cannot be fully explained by ocular examination alone [7,8]. These disabilities have been shown to persist throughout an individual’s life [9–11]. CVI resulting from early onset brain injury still lacks a universally accepted definition. Initially, CVI was described as a visual dysfunction not attributable to issues with ocular or anterior visual pathways [7]. More recently, a report from the National Institutes of Health CVI Workshop proposed a more expansive working definition encompassing 5 key components of CVI, the last of which emphasizes that CVI can often remain “unrecognized or undiagnosed” until later stages of life [8]. However, most of our current knowledge of CVI is derived from studies of children; adults with early-onset CVI have not been systematically studied to determine the pattern of visual loss and HVFDs in adults over time.

### 1.1. Cerebral visual impairment in children

Early-onset CVI (C–Early Onset) has been well described in children and adolescents. It typically results from retrochiasmatic brain lesions linked to adverse events occurring at or around birth and is also associated with brain development disorders. Among the various etiologies, perinatal hypoxic-ischemic encephalopathy (HIE) is recognized as the most common underlying cause of CVI [7,12–15]. Other causes are hydrocephalus, meningitis and encephalitis, metabolic, structural brain abnormalities, in-utero drug exposure [16–21] and/or genetic disorders [22,23].

VA may vary from normal to profound loss. HVFDs are common and sometimes the only symptom complex in children with good VA. They encompass deficits in spatial visual perception, visual cognition, visual attention, motion perception, and visual guidance of movement. The neural mechanisms involved in this process reflect the high complexity of brain architecture and the multifaceted relationships between vision and other sensory and motor domains. Therefore, diagnosing HVFDs is essential since they can cause significant visual disability in everyday activities and education, even if VA remains largely intact [1,3,4,14,24–26]. They may present with eye movement disorders and accommodative dysfunctions [27].Children with CVI are often not aware of their HVFDs, showing no ophthalmological complaints [28]. An increasing awareness of CVI in children is slowly leading to a greater understanding of the manifestations, implementation of habilitation measures, provision of support and resources needed at home, in the community and in education [29]. However, the visual impairments in adults with long-standing early-onset CVI may change in their severity and be affected by the different challenges of an adult visual environment, independent living, employment, travel and social interaction.

### 1.2. Adults with early-onset CVI (A–Early Onset)

The only systematic controlled study of HVFDs in adults with early-onset brain injury has focused solely on a single higher visual function, where adults with Williams syndrome reported difficulties with global motion perception, though they were not classified as CVI [9]. Over time, studies in adults with early-onset CVI have concentrated on assessing visual and oculomotor function [30–32] with only a single case study tracking changes in higher visual functions beyond visual function over a six-year time period of childhood showing the efficacy of intervention strategies [33]. In a separate longitudinal study involving children with cerebral palsy and CVI, findings showed that some visual functions (VA, contrast) and oculomotor functions (smooth pursuit; saccades) improved over childhood while others declined. A sub-cohort (51/63) analysis revealed an association between cognitive visual dysfunction (unspecified IQ tests) and oculomotor abnormalities [34]. Adults with early onset CVI also exhibited reduced white matter integrity in both dorsal and ventral white matter pathways, potentially contributing to observed impairments in visual perception and function [35]. However, a cross-sectional or longitudinal cohort study exploring the progression of functional visual impairments in CVI over time has not yet been studied. Our cross-sectional research examining HVFFDs in older children [3,4] provides compelling evidence that deficits in visual acuity and HVFDs persist into adolescence (sub-analysis of a cohort of 10–18 years of age, n = 44, mean±SD: spectrum 0.65 ± 0.26; severity 3.11 ± 0.86, significantly higher than a sub-analysis of a cohort of NTs of similar age group). However, our knowledge of HVFDs in adults with early-onset CVI and its functional vision challenges remains limited to those reported and described by individuals with CVI to investigators [36,37] of a particular syndrome [9] or at CVI support websites such as CVI Scotland and CVI NOW. These limited studies, recent personal published perspectives of children and adult CVIers [5,38] and our own review of our cohort of older children indicate significant challenges with HVFDs persisting in adult life even in the presence of near-normal VA, underscoring the urgent need for a comprehensive, systematic study in this population.

### 1.3. Adults with late-onset brain injury (A–Late Onset)

Six percent (6%) of the adult population may develop a neurological condition over the course of their lifetime. Late-onset brain injury in adults can result from a range of acute events such as stroke or trauma, as well as chronic conditions including neurodegenerative diseases, infections, and the effects of long-term substance abuse or metabolic dysfunctions. Late-onset brain injury presents an underlying pathophysiology not dissimilar to early-onset CVI, though one occurs in a mature brain while the other occurs in an immature developing brain. Nevertheless, for the majority of adults with CVI, the origin lies in local brain damage (*e.g.,* traumatic brain injury and stroke) that can be reliably identified through imaging [39]. The motor and sensory effects of late-onset brain injury in adults have been widely studied in the literature, particularly in relation to stroke or traumatic brain injury [40]. Historically, the effects on visual fields and visually mediated spatial perception and recognition from intracranial cerebral injuries have also been documented through landmark detailed case reports [41,42] and review articles [43,44]. Visual impairments reported following the most prevalent cause of late-onset brain injury (a stroke) have predominantly focused on a study of visual function, such as: impaired VA; visual field loss, ocular motility disorders, and visual inattention [45,46]. Stroke has been studied extensively, leading to the identification of a set of core outcomes for visual function, functional vision, and ocular motility after a stroke [47]. When VPDs, such as visuospatial disturbances; agnosia, akinetopsia, and aphasia [47–49] are described or assessed in late-onset brain injury, they are not dissimilar to the HVFDS in CVI. However, they are not commonly assessed for A–Late Onset [50]. A qualitative study investigating current practices for screening visual perceptual deficits in adults post-stroke, based on interviews with healthcare professionals in England [51], revealed limited awareness of these deficits among professionals. This highlights a clear demand for enhanced training and the need for an ideal standardised screening tool for assessment of VPDs for brain-injured patients.

Although the terms CVI and HVFDs have been widely used in the context of early-onset CVI, they have not been consistently applied to describe the neurological condition or the visual impairments seen in A–Late Onset. Instead, visual perceptual deficits [47,51] and cognitive impairment [52] are more commonly used to describe HVFDs. Visual impairments are textually embedded within broader diagnostic labels such as stroke and traumatic brain injury. The NEI website on CVI in children notes that the problems seen in adults from brain injury is “sometimes called Acquired CVI”, “not the same as CVI”, and “usually has different symptoms than CVI” [53]. We have chosen not to use the term Acquired CVI, as early-onset CVI typically results from adverse events at birth while late-onset CVI emerges from adverse events occurring in adulthood, and thus all are “acquired”. Moreover, the term Acquired CVI for all CVIers (adults and children) as separate from genetic causes of CVI has been used in several articles, in contrast to the definition at the NIH website [27,54]. Given the lack of consensus around terminology and to prevent ambiguity, we will use the term A–Late Onset when necessary, referring to Adults with Late Onset Brain Injury.

### 1.4. Comparison A–Early Onset and A–Late Onset

In the absence of direct comparative studies, only indirect comparisons can currently be made between the outcomes of early and late brain injury. Dutton explored the similarities and differences in higher visual function deficits in clinical presentations with examples of case histories in adults and children with brain injury using the 2-stream model of dorsal and ventral streams [55]. Additional insights come from the studies of visual deficits in children, adolescents, and adults with Traumatic Brain Injury (TBI) from non-accidental injury soon after birth to falls in the elderly. Across these populations, significant HVFDs affecting spatial perception and visual field deficits and oculomotor dysfunctions have been observed [10,56–60]. Studies of TBI in children, beyond the early developmental years, often demonstrate more promising recovery due to heightened neuroplasticity. Conversely, injuries occurring in very early developmental periods (birth to a year or less) may not show such recovery; the reasons being unclear with theories that early onset brain injury interferes with development of immature or rapidly developing skills and, may be associated with further magnification of deficits during later development [61–63]. In adults, chronic impairments from TBI are more common, with slower recovery trajectories and a greater risk of permanent deficits [64]. Although the impact of age differences in TBI outcomes is well-documented, there have been no studies directly comparing the spectrum and severity of HVFDs in adults with early-onset versus late-onset brain lesions. This represents a critical research gap. Overall, while both early and late-onset injuries affect similar neural substrates, it remains unclear whether the clinical outcomes truly differ between the two. Injuries in a developing brain may produce more diffuse damage versus limited loss of visual function from the typical discrete injury in a mature brain. The opposite may apply to reorganization of the brain for restoring impaired motor and sensory function, including visual abilities. Perinatal hypoxia, the leading cause of EOCVI, often affects several brain regions, whereas A–Late Onset, caused by the commonly circumscribed lesions such as tumors and infarcts, may result in localised sensory and motor deficits correlating with the damaged area. Additionally, children with EO-CVI with impaired visual development due to early injury are most likely agnostic of normal visual function, whereas adults with late-onset brain injury will have lost discrete aspects of previously held visual abilities. Adult-onset brain injury has not been formally categorized as CVI, and the reasoning behind this remains unclear. It is possible that the separation stems from the knowledge of adult late-onset brain injury and indirect comparison with children with early-onset CVI but not of the adult with early-onset CVI. There remains a substantial gap in our understanding of similarities and differences in the adult with early versus late onset brain injury, which likely has implications for assessment and interventions tailored to these two groups of patients.

#### 1.4.1. Neural Basis of early-onset CVI originates from studies of late-onset brain injury.

Although the neural basis of HVFDs in early-onset CVI is frequently interpreted based on damage within the 2-stream model – the dorsal stream and ventral stream of visual processing – much of this framework is derived from studies of primates reviewed in a book chapter [65] and studies involving late-onset brain injury [66,67], comparative primate studies, and adult late-onset human lesion studies [68].

Briefly, the anatomical substrates in the occipito-parietal lobes (dorsal stream) and occipito-temporal lobes (ventral stream) pathways are associated with the superior and inferior longitudinal fasciculi, respectively. The dorsal stream, responsible for non-conscious visual mapping of the surroundings (the “where” pathway), and the ventral stream, responsible for recognition (the what pathway) are well described, and the two pathways likely interact through the vertical occipital fasciculus – the connection between the dorsal and ventral streams [69,70] emerged in recent years as a functionally active structure. Damage to either of these two-streams can give rise to HVFDs.

Damage to the dorsal stream serves as a non-conscious visual function being the primary mediator for HVFs such as automatic, non-conscious visual guidance of movement, and the management of complex visual scenes [66,71]. In contrast, ventral stream lesions affect HVFs, such as conscious perception and cognition, leading to deficits in object, word, and face recognition [72]. Classic historical studies of higher visual function disorders in eponymous syndromes (Anton, Charles Bonnet and Balint syndromes) are described in adults with late-onset brain injury and shaped current understanding of visual processing and show considerable overlap with the manifestations of CVI reported in more recent literature, as summarized in a recent review and update [73].

In recent years, growing evidence from advanced brain imaging methods has begun to shed light on the typical development of the dorsal and ventral visual streams, with studies now extending to children as young as 4 years and drawing comparisons with adult brain organization (for review see Klaver et al., 2011). Of particular interest are the interactions facilitated by the posterior vertical pathway (PVP), where Vinci Boonar et al [74] propose an input-driven model. In early-onset CVI, structural and functional brain imaging in adolescents and young adults with CVI revealed evidence of anomalous development of dorsal and ventral streams with correlation to functional vision impairments, characteristic of early-onset CVI [75–78].

Direct comparisons with structural and functional studies using current methods between early and late-onset brain injury would provide contemporary evidence for similarities and differences and add to our understanding.

### 1.5. The study

In this prospective controlled study, we characterize Higher Visual Function Deficits (HVFDs) in adults with early-onset Cerebral Visual Impairment (A–Early Onset) and compare them to the pattern of HVFDs in adults with late-onset brain injury (A–Late Onset) and adult neurotypical controls (ANT). Additionally, we compare the HVFD patterns between the two adult cohorts (A–Early Onset and A–Late Onset) with a cohort of children with early-onset CVI (C–Early Onset) from our previous studies (including child neurotypicals (CNT)) [3,4]. All three adult cohorts were assessed using the Higher Visual Function Question Inventory (HVFQI), a semi-structured tool designed to elicit HVFDs in CVI [3,4]. The primary aim of this study was to assess the reliability of the rephrased Adult Higher Visual Function Questionnaire in eliciting HVFDs in A–Early Onset. The secondary aim was to characterize the spectrum and severity of HVFDs in this population and to compare these patterns with those observed in C–Early Onset investigated in our previous studies [3,4]. The third aim involved including a cohort of A–Late Onset to compare the spectrum and severity of HVFDs between individuals with A–Early Onset and those with A–Late Onset, in order to better understand if the timing of the onset of brain injury affects the nature of HVFDs.

Our hypothesis for the primary aim was that the rephrased Adult HVFQI will demonstrate similar reliability to the 4–18-year-old version of the HVFQI in assessing HVFDs in adults with CVI. Additionally, we expect that HVFDs in adults with AEO-CVI will persist over time but will present a distinct pattern due to the long-standing nature of the condition, as well as the challenges of navigating an adult life and environment. This pattern may also have been influenced by the development of strategies to cope with or mask these deficits over time. Finally, we hypothesize that the pattern of HVFDs would differ between individuals with early-onset and late-onset CVI. This difference may arise due to the distinct ways in which brain structure and function are affected in the developing brain (with more diffuse lesions) compared to the mature brain (which may be impacted by more localized lesions). However, we acknowledge that the differences may not always be clear-cut, and some overlap in the patterns of visual deficits may exist between these two groups.

## 2. Methods

Participant Recruitment and Consent: Participants for this study were prospectively recruited between February 2022 and October 2024. Informed consent was obtained from all participants prior to inclusion in the study. Consent was collected via written documentation, which was electronically signed through DocuSign, a secure digital signature platform. This process ensured that participants were fully informed about the nature of the study, the procedures involved, and any potential risks. The ethical approval for the study CHA002 was granted by the Institutional Review Board Ethics Committee, Smith-Kettlewell Eye Research Institute. Adult data were collected prospectively and paediatric data came from a previously published cohort [4]. Additional information regarding the ethical, cultural, and scientific considerations specific to inclusivity in global research is included in the Supporting Information (S1 Checklist).

### 2.1. Adult Higher Visual Function Question Inventory (HVFQI-59Q)

#### 2.1.1. Instrument structure and scoring.

The Higher Visual Function Question Inventory (HVFQI) is a semi-structured inventory designed to elicit behavioural evidence of higher visual function deficits (HVFDs) associated with cerebral visual impairment (CVI). The HVFQI was modified from the CVI Inventory (CVI-I) as previously described [3,10,79], and was administered using the HVFQI Web App during structured remote video interviews [80]. For the present adult study, the inventory was adapted from the paediatric HVFQI without changing its core functional content. The original HVFQI-51 contained 51 questions [3], and 7 additional questions were incorporated in the HVFQI-58Q used in Chandna et al., 2024 [4]. The adult version used here, the HVFQI-59Q, was adapted from the HVFQI-58Q with the addition of one further question. A fuller description of the paediatric-to-adult adaptation is provided in Section 2.1.2.

The adult HVFQI-59Q contains 59 items and is designed to assess higher visual functions commonly affected in brain-based visual impairment. Within the Web App, items are organised in clusters for practical administration and interpretation, including questions relating to spatial perception, navigation, visual attention in complex scenes, motion-related judgements, clutter and crowding, object and face recognition, reading-related visual difficulties, and visually guided actions. These clusters reflect functions often discussed within dorsal-stream-weighted and ventral-stream-weighted frameworks [55,66–68]. However, the instrument does not assume strict neurobiological separability of domains, because many visually guided behaviours recruit distributed and interacting cortical systems spanning dorsal and ventral pathways as well as frontal and multisensory networks. Accordingly, the primary quantitative analyses in this study were based on item-level responses rather than on presumed neurobiological domain boundaries.

Responses were recorded using a 5-point Likert scale: Never, Rarely, Sometimes, Often, and Always, scored from 1 to 5 respectively, with higher scores indicating more frequent difficulty. Two additional response options were available: Not Applicable, for behaviours not relevant to the participant’s circumstances or not interpretable because of comorbid conditions, and Not Assessed, for behaviours with which the participant was insufficiently familiar to provide a meaningful answer. These responses were excluded from scoring calculations, and the reason for their use was documented during the interview.

Questionnaire-derived summary measures were calculated from item-level responses. The HVFD Severity Index was defined as the mean scored response across included items, using the 1–5 Likert coding described above, after excluding Not Applicable and Not Assessed responses. Higher severity values therefore indicate more frequent reported visual difficulty across the questionnaire. The HVFD Spectrum Index was defined as the proportion of items meeting the threshold for an impactful deficit after excluding Not Applicable and Not Assessed responses. As in prior HVFQI work [3,4], the threshold for deficit endorsement was determined empirically. In the adult cohort, the cutoff of Sometimes or higher (i.e., Sometimes, Often, or Always) provided the greatest separation between CVI and neurotypical response distributions; the derivation and justification for this adult cutoff are described in Section 3.1.1.

#### 2.1.2. Adaptation from the paediatric HVFQI to the adult HVFQI-59Q.

The adult HVFQI-59Q was derived directly from the previously published paediatric HVFQI-51 [3] and the extended HVFQI-58Q [4]. The adaptation was undertaken as a contextual modification for adult use rather than as the construction of a new instrument. The underlying behavioural constructs, response format, scoring framework, and analytic approach were retained from the earlier HVFQI versions, with modifications focused primarily on wording, examples, and ecological relevance for adult life.

The adaptation process had four main features. First, the core constructs assessed by the HVFQI were preserved, including difficulties relating to spatial perception, visual search, motion perception, clutter and crowding, navigation, recognition, and visually guided action. Secondly, questions were rephrased for age-appropriate adult self-report and adult functional contexts. In particular, school-based or child-centred examples were reframed to reflect adult demands such as independent navigation, use of public transport, screen- and document-based tasks, workplace or community environments, and more complex social and visual scenes. Thirdly, the scoring structure was unchanged, such that the adult version remained directly comparable at the item-response level with earlier HVFQI work. Fourthly, no construct-defining domains were removed during adaptation. The single-item difference between the HVFQI-58Q and the adult HVFQI-59Q arose from adult adaptation of item content, while preserving the overall framework of the instrument.

The adult-adapted wording was developed and reviewed in structured workshops involving adults with CVI, clinicians, researchers, and teachers of the visually impaired with experience in CVI, in order to improve clarity, face validity, and ecological validity in adult contexts. These workshops focused on whether the questions were understandable in adult self-report form and whether the examples reflected real-world adult visual demands without changing the intended behavioural construct.

Examples of rephrased items are provided in the S1 Appendix. A structured documentation of the principal paediatric-to-adult adaptations is also provided to document items that were reworded, expanded, or otherwise substantively modified for adult use. Because the adult HVFQI-59Q preserves the core behavioural constructs and scoring approach of the earlier HVFQI instruments, it should be regarded as a contextual adult adaptation of the existing questionnaire framework rather than a wholly new instrument.

#### 2.1.3. Preliminary psychometric performance of the adult HVFQI-59Q.

The psychometric performance of the adult HVFQI-59Q was examined in the present adult cohort, with particular attention to internal consistency and score distribution in the adult neurotypical group. Discriminative performance of the adult instrument is reported in Sections 3.1.1 and 4.1, where the adult spectrum cutoff was evaluated using median separation (Δ median) and root mean square (RMS) comparisons between CVI and neurotypical participants across alternative response thresholds. For direct comparison with earlier paediatric work, the adult inventory was also mapped to the shared 51-item set by excluding items 52–59, as described in the Analysis section.

Internal consistency of the adult HVFQI-59Q total score in the adult neurotypical cohort was high (Cronbach’s alpha = 0.94), indicating strong coherence across items. To assess continuity with the previously validated paediatric instrument, internal consistency was recalculated after restricting the adult questionnaire to the shared 51-item subset corresponding to the original HVFQI-51 [3]. This 51-item subset also showed high internal consistency (Cronbach’s alpha = 0.93), supporting structural continuity between the adult-adapted instrument and the earlier paediatric version.

Inspection of score distributions did not indicate problematic floor or ceiling effects that would limit discriminative capacity. In the adult neurotypical cohort, responses clustered toward the lower end of the scale, as expected, with only 3/67 participants achieving the minimum possible mean score and none achieving the maximum. In the adult neurologically impaired cohort, no participant achieved the maximum possible mean score, indicating no evidence of ceiling compression. Missing item-level data in the adult neurotypical cohort were minimal, with 14/3953 item responses coded as Not Applicable or Not Assessed and therefore excluded from scoring calculations.

The previously validated HVFQI-51 demonstrated high internal consistency and reliability in children [3]. The adult HVFQI-59Q preserves the same core item framework, Likert response structure, handling of Not Applicable and Not Assessed responses, and the same analytic derivation of severity and spectrum indices. Taken together with the discriminative performance demonstrated in the present adult sample, these findings support the adult HVFQI-59Q as a reliable and interpretable instrument for characterising HVFD-related difficulties in adults.

#### 2.1.4. Recruitment.

Study participants were recruited through clinical institutions, educational networks, CVI support groups, information websites, and social media groups, parents, teachers of the visually impaired, colleagues, and friends. Recruitment was voluntary, and participants self-selected after reviewing the study information. No explicit targeting based on symptom severity or functional level was employed. Following an expression of interest in the research project by the CVIer or caregiver, research information, consent, and assent documents were provided for review. A face-to-face virtual meeting (Zoom) was set up across time zones at a mutually convenient time. At the meeting, informed digital e-consent was obtained, which included the sharing of clinical information. Assent was also obtained where necessary if the interview was conducted with a caregiver of an adult CVIer. All participants in the neurologically impaired groups had a prior clinical diagnosis of CVI or brain injury. None had completed the HVFQI previously, and most had not undergone a formal functional visual assessment.

The HVFQI-59Q was administered in an interviewer-led format during remote Zoom interviews by trained study personnel (AC and SV) using the HVFQI Web App. Questions were presented in a standardised manner, and participants selected their own responses independently. Non-leading clarifications were provided only if a question was not understood. Once a response was recorded and the next question presented, the response could not be changed. Administrators were aware of participant group allocation; blinding was not implemented. To minimise inadvertent influence, interviewers followed standardised procedures and used non-leading explanations throughout.

#### 2.1.5. The adult HVFQI interview.

The interviews via Zoom commenced with standardized instructions. A unique participant ID was generated. Single, randomly generated questions were displayed on a shared screen to the participant along with the range of possible responses (the Likert scale and the Not Applicable options). The adult CVIer or the caregiver was encouraged to read the question and choose one option. If requested, questions were clarified with non-leading explanations. Comments from the parent were noted in the free text section on the screen. If the Not Applicable or Not Assessed option was chosen, the reason was documented. The HVFQI-59Q was completed in one session, and within-session breaks were given if necessary. We did not control the response time. The overall range of time taken was between 6–67 minutes (mean ± SD: 31.15 ± 13.45 minutes, n = 61). The average questionnaire response time for CVI participants took twice as long (33.60 ± 13.17 minutes, n_CVI_ = 51) than for Neurotypical (NT) participants (15.10 ± 13.32 minutes, n_NT_ = 9).

### 2.2. Participants

Our study comprises 51 adults with early-onset (n = 29) CVI and late-onset (n = 22) brain injury and adult neurotypicals (n = 67). We compare our findings in adults with 92 children with CVI and 120 neurotypical children from our previous study cohort [4].

#### 2.2.1. Participant demographics, visual acuity, birth conditions, and other clinical information.

Patient demographics and visual acuity summaries are provided in Table 1, including sex, age, and binocular visual acuity (VAOU). Years of education were not systematically collected and were therefore unavailable for analysis. Information on participant birth prematurity can be seen in Table 2. For each adult participant, details on etiology, MRI brain imaging findings, and comorbidities for adults with early-onset CVI are presented in Table 3, while information for adults with late-onset brain injury is found in Table 4.

**Table 1:** Participant characteristics.

Age Group	Cohort	Count	Gender (F/M/O)	Age (Mean±SD)	VAOU (LogMAR)
Adults 17–70	A–Early Onset	29	59% / 38% / 3%	36.9 ± 15.8	0.27 ± 0.34
	A–Late Onset	22	68% / 32% / 0%	29.33 ± 10.2	0.12 ± 0.30
	ANT	67	66% / 34% / 0%	47.0 ± 16.5	0.01 ± 0.03

**Table 2:** Adult participant preterm birth (World Health Organization classification).

Cohort	At-Term	Moderately Preterm	Very Preterm	Post Term	Unknown
A–Early Onset	14 / 42.3%	6 / 20.7%	3 / 10.3%	1 / 3.5%	5 / 17.2%
A–Late Onset	20 / 86%	0 / 0%	0 / 0%	0/0%	2 / 13.6%
ANT	60 / 89.6%	4 / 6%	1 / 1.5%	2 / 3%	0 / 0%

Abbreviations used: Adults with early-onset CVI (A–Early Onset); Adults with late-onset Brain Injury (A–Late Onset); Adult Neurotypicals (ANT)

**Table 3:** Primary diagnosis and brain-MRI scan results of adults with early onset CVI.

ID	Primary Diagnosis	Brain MRI findings	Comorbidities
1	Dup 15 q chromosome duplication syndrome	Not available	Epilepsy
2	KCNH1 gene mutation syndrome	Microcephaly	CP, Epilepsy
3	HIE	Normal	CAPD
4	HIE	Normal	CAPD
5	HIE	Cortical atrophy	CP
6	Methylene THF reductase deficiency	Not available	ASD
7	HIE	Normal	CP, ADHD
8	HIE	Cortical atrophy	CP, CAPD
9	Hydrocephalus	Enlarged ventricles	ASD, ADHD
10	Metabolic Syndrome	Normal	Dyslexia, Epilepsy
11	HIE	Not available	Dyslexia, Apraxia, ASD, ADHD
12	Hydrocephalus	Enlarged lateral ventricles	
13	Newborn symptomatic Hypoglycemia	Not done	
14	Neonatal Infection	Cortical atrophy	ASD, CP
15	Hydrocephalus	Enlarged third and fourth ventricles	ASD
16	Down Syndrome	Not done	ASD
17	Neonatal Infection	PVL bilateral	ASD
18	Neonatal Hemorrhagic Stroke	PVL	CP
19	Neonatal Hemorrhagic Stroke	PVL Right Temporal and Parieto- Occipital	ASD, CAPD
20	Neonatal meningitis	Bilateral Occipital ischemia	
21	CTX Syndrome (Cerebro-tendinius xanthomatosis)	PVL parieto- occipital and Corpus Callosum abnormality	
22	Genetic Syndrome	Cortical atrophy	CP, ASD, ADHD, Epilepsy
23	HIE	PVL	CP
24	Malan Syndrome	Normal	ASD
25	Malan Syndrome, Global Developmental Delay	Macrocephaly and Pituitary gland abnormality	
26	Malan Syndrome, Global Developmental Delay	PVL	CP
27	Malan Syndrome, Global developmental delay	Macrocephaly	
28	Malan Syndrome	Corpus Callosum Dysplasia	ASD
29	Malan Syndrome, Global Developmental Delay	Normal	Apraxia

Abbreviations used: Autistic Spectrum Disorder (ASD); Attention Deficit Hyperactivity Disorder (ADHD); Hypoxic Ischemic Encephalopathy (HIE); Periventricular Leukomalacia (PVL), Cerebral Palsy (CP), Central Auditory Processing Disorder (CAPD).

**Table 4:** Neurological diagnosis and brain-MRI scan results of adults with late-onset brain injury (A–Late Onset).

ID	Primary Neurological Diagnosis	Brain MRI findings	Comorbidities
1*	*Brain Tumor*	*Right Frontal Lobe Glioma*	
2	Stroke	Bilateral Occipital Lobes infarct	
*3*	*Stroke*	*Left Frontal Lobe infarct*	
4	Stroke	Right Occipital Inferior Parietal Lobe infarct	
5	Mild Traumatic Brain Injury	Normal	
6	Mild Traumatic Brain Injury	Normal	
7	Myasthenia Gravis	Right Cerebellar Nodule	CAPD, Hearing loss
8	Stroke	Cerebellum and Occipital lobes infarcts	Epilepsy
9*	*Stroke*	*Left Inferior Occipital lobe infarct*	
10	Meningitis, Stroke	Left Thalamus and Occipital lobe	ASD, CP, ADHD
11	Traumatic Brain Injury	Dilatation of lateral ventricles and bilateral Occipital Lobe hemorrhage	Epilepsy, Aphasia
12	Stroke	Midbrain Stem infarct	
13	Alzheimer disease early stage	Posterior Cortical Atrophy	
14	Meningitis, Multiple Sclerosis	Not available	
*15*	*Mild Traumatic Brain Injury*	*Not available*	
16	Traumatic Brain Injury	Left Temporal Lobe damage	
17	Traumatic Brain Injury	Frontal and left Temporal Lobe damage	
18	Traumatic Brain Injury	Normal	
19	Stroke	Right inferior Frontal Lobe and Inferior Temporal-Occipital Lobes infarcts	
20	Benson’s Syndrome	Posterior Cortical Atrophy	
21	Brain tumor	Pituitary Tumor	
22	Stroke	Right Occipital Lobe Infarct	

Abbreviations used: Central Auditory Processing Disorder (CAPD); Autistic Spectrum Disorder (ASD); Attention Deficit Hyperactivity Disorder (ADHD). Note: We will discuss participants #1, 3, 9, 15 in greater depth in sections 4.3 and 5.2, as they are outliers in Fig 2A).

#### 2.2.2. Adults with early-onset CVI (A–Early Onset).

Diagnosis of early-onset CVI was established following a detailed history including the course of pregnancy; details of adverse events at or around birth; developmental and family history; eye and vision history; review of clinical notes including most recent eye examination; brain imaging and report/s stating diagnosis of CVI.

#### 2.2.3. Adults with late-onset brain injury (A–Late Onset).

Diagnosis of late-onset brain injury was established following a detailed history regarding the brain injury event and course with relevant treatment. History was also obtained regarding the course of pregnancy; any adverse events at or around birth; developmental and family history, and if not certain, the gaps were completed with efforts made by the participant or caregiver through family members. A detailed eye and vision history was obtained; we undertook a review of clinical notes, including the most recent eye examination and brain imaging, and report/s stating a diagnosis of the event and consequences of the brain injury.

#### 2.2.4. Adult neurotypical group (ANTs).

A diagnosis of neurotypical was made following a detailed history excluding any adverse events at or around birth; genetic conditions; developmental and family history; general health status; and any previous or current neurological events and any current or past medications. If not certain, the gaps were completed with efforts made by the participant through family members. A detailed eye and vision history to exclude any visual impairment, confirmation of normal visual acuity and clinical notes and eye examination reports were reviewed, if relevant.

### 2.3. Comparison with CVI (and NT) cohorts in children

We compare the adult CVI and NT cohorts with the child CVI and NT cohorts and describe them briefly below. For details see [4]. For comparison with the previously published paediatric cohorts [4], adult and paediatric HVFQI data were harmonised onto the shared 51-item set corresponding to the original HVFQI-51 [3]. Specifically, responses from the adult HVFQI-59Q and the paediatric HVFQI-58Q were mapped to the common 51-item core, and the HVFD Severity and HVFD Spectrum indices were recalculated on this harmonised basis. Adult–child comparisons were therefore based on harmonised indices derived from the shared item set, rather than on raw equivalence of the full adult and paediatric questionnaires. This approach preserved continuity of the core behavioural constructs across studies while recognising that some differences in wording, response framing, and developmental context remained and should be considered in interpretation.

#### 2.3.1. Children with early-onset CVI group (C–Early Onset).

For comparison, we included the cohort from our previous work: 92 children with CVI (42 female, 50 male), mean ± SD age (9.4 ± 3.6 years) with VA (0.26 ± 0.29 logMAR). with an established diagnosis of CVI [4].

#### 2.3.2 Neurotypical Children Group (ChNTS).

The neurotypical group (NT) of 120 typically developing children (from [4]), mean age 7.2 ± 2.8 years and VA of 0.0 ± 0.0 logMAR.

## 3. Analysis

We followed previously published methodologies to calculate the **HVFD Spectrum Index** (spectrum) and **HVFD Severity Index** (severity) for each participant in both the neurologically-impaired and neurotypical cohorts. These distributions were compared visually via box-and-whisker plots and statistically using the Mann-Whitney U test [3,4]. These distributions were compared visually using box-and-whisker plots and statistically using two-sided Mann–Whitney U tests (Wilcoxon rank-sum tests) for the pre-specified pairwise comparisons described below. A prespecified significance threshold of α = 0.00001 was used. No formal correction for multiple comparisons was applied, because the principal comparisons were pre-specified and limited in number; exact p-values are reported throughout to allow direct interpretation of the strength of evidence.

Age, sex, and VAOU were considered as potential confounders in interpreting between-group comparisons. However, the primary analyses were pre-specified non-parametric comparisons of derived HVFD indices, and covariate-adjusted modelling was not applied because this was an exploratory study with modest subgroup sizes and incomplete covariate information, including the absence of systematically collected education data.

### 3.1. HVFD severity and spectrum indices

The HVFD Severity Index is calculated as the mean response to the HVFQI, where each individual response is assigned a numeric score ranging 1–5: 1 (*Never*), 2 (*Rarely*), 3 (*Sometimes*), 4 (*Often*), and 5 (*Always*). *Not Applicable* and *Not Assessed* responses are excluded.

The HVFD Spectrum Index measures the diversity of visual deficits exhibited by an individual with CVI. It ranges from 0 (no impairments) to 1 (expression of all impairments measured). For example, if a participant reports impactful visual deficits for 44 out of 51 questions, their Spectrum Index is 44/51 = 0.863. Responses marked *Not Applicable* or *Not Assessed* are excluded before this calculation. The next section explains how we determine which response threshold indicates impactful visual deficits.

#### 3.1.1. Choice of HVFD spectrum index cutoff for the adult HVFQI.

In Chandna et al., 2024, the responses of children aged 4–18 years (n_Total_ = 144, n_CVI_ = 92, n_NT_ = 120) were separated into five dichotomies at different levels of expression. In that study, the cutoff threshold of *Sometimes* or higher (i.e., *Sometimes, Often, Always*) was determined to provide the maximum difference between the median scores (Δ median) between the response distributions of CVIers and neurotypicals. Accordingly, the minimum level of expression for a visual deficit to be considered for the spectrum calculation for children is *Sometimes*.

In this study, we evaluated the choice of spectrum cutoff for the HVFQI-59Q instrument designed for adults. In adults (n_Total_ = 118, n_CVI_ = 51, n_NT_ = 67), the *Sometimes* response cutoff threshold provides the largest separation between the CVI and NT cohorts (Δ median: *Rarely*: 0.53*,* ***Sometimes*: 0.66***, Often*: 0.51*, Always*: 0.30). This finding was confirmed using root mean square (RMS) between pairwise responses of CVI and NT participants (RMS: *Rarely*: 0.53*,* ***Sometimes*: 0.59***, Often*: 0.49*, Always*: 0.35). We can directly compare the 59Q inventory used for adults with the 2021 51Q inventory used for children by filtering responses for questions 52–59. When converted, the *Sometimes* response cutoff still provides best separation between dichotomies (Δ median: *Rarely*: 0.54*,* ***Sometimes*: 0.63***, Often*: 0.49*, Always*: 0.26) and confirmed with RMS: (RMS: *Rarely*: 0.54*,* ***Sometimes*: 0.59***, Often*: 0.49*, Always*: 0.34).

### 3.2. Investigations

In this study, we addressed four fundamental questions: (1A) Are there differences between the spectrum and severity distributions of adult early-onset CVI (A–Early Onset) versus adult neurotypicals (ANT)? (1B) Adult late-onset brain injury (A–Late Onset) versus ANT? (2) Are there differences between adult early-onset CVI versus child early-onset CVI (C–Early Onset)? (3) What are some of the emerging characteristic differences of mean responses from the A–Early Onset versus A–Late Onset groups? (4) What are the relationships between spectrum and severity for A–Early Onset, A–Late Onset, and ANT, and how do they compare with the linear relationship for C–Early Onset and CNT groups found in Chandna et al., 2024?

#### 3.2.1. Comparing HVFD indices for adult early-onset CVI (A–Early Onset) vs adult neurotypicals (ANT) and adult late-onset brain injury (A–Late Onset) vs. adult neurotypicals.

Data collected using the HVFQI-59Q for 18 + adults were extracted from the HVFQI Web App database and all calculations with adult cohorts were made using all responses. Comparisons with A–Early Onset, A–Late Onset, and adult neurotypicals were also made using the responses to the full 59 question inventory.

Plotting HVFD spectrum and severity distributions using box-and-whisker plots allows for clear visual comparisons between adult CVI and NT groups. For each metric, the Mann-Whitney U-Test (MWU test) can then assess whether A–Early Onset vs. ANT and A–Late Onset vs. ANT results are likely drawn from the same distribution (the null hypothesis). For this study, we chose a strict *p*-value cutoff (ɑ) of 0.00001; a *p*-value lower than ɑ informs us to reject the null hypothesis. If the null hypothesis cannot be rejected, it suggests no significant difference in HVFDs between CVI and NT. Conversely, rejecting the null hypothesis indicates that these indices effectively detect HVFD differences between CVI and NT spectrum or severity distributions.

In section 1, we hypothesized that HVFD acquisition later in life would have differing patterns of HVFDs when compared with CVI as a result of perinatal conditions. We test this hypothesis by looking to see if those effects (and effects like them) can be found in the data by visually comparing distributions using box-and-whisker plots and calculating the likelihood of HVFD differences using the MWU test.

#### 3.2.2. Comparison of HVFD expression in CVI amongst adults and children.

Data collected using the HVFQI-58Q for 4–18-year-old children and the HVFQI-51Q [3] were mapped to the 51 questions inventory to allow direct comparisons of adult responses with child responses. The mapping from the 59Q and 58Q to the 51Q inventories is direct and linear, with some loss of information (7–8 questions). There is some redundant coverage between the lost questions and preserved questions, so the overall information loss is approximately 4–8%, which is acceptable loss to bring the larger data samples into analysis and to be able to build upon prior work.

Visual comparison with box-and-whisker plots can establish high-level differences in HVFD expression between adults and children with CVI, if any exist. The MWU test can also be used to identify differences in HVFD metrics between adults and children with CVI. Similar to the previously described CVI versus NT comparison, the test is conducted for both Spectrum and Severity indices, but this time to compare CVI in adults against CVI in children. Scatterplots of HVFD spectrum versus severity show not only the relationship between spectrum and severity in adults and children but also differences in that relationship between adults and children as well. For example, given that CVI can be caused by head injury, lesions, or other localized insults experienced after vision has fully developed, we might expect CVI in some adults to manifest as high severity among a low number of visual deficits, as opposed to more widespread effects from perinatal hypoxic-ischemic encephalopathy. As another, complementary example, adults who have had CVI their entire lives may have learned to effectively employ compensation strategies to best utilize their limited functional vision to overcome visual function deficits. This might show as an overall attenuated severity among adults, even if there is a large spectrum of visual deficits.

#### 3.2.3. Characterizing differences in mean response between HVFDs in A–Early Onset, A–Late Onset, and C–Early Onset.

To explore potential trends between brain-based visual impairment acquisition modes A–Early Onset, A–Late Onset, and C–Early Onset, we plotted the mean response for each question in stacked column graphs for both the adult (59Q) and child (51Q) inventories. Comparisons were made against neurotypical peers. While some observed differences may reflect age-related interpretation of questions, consistent patterns could point to age- or onset-specific features of CVI expression that merit further study.

#### 3.2.4. Comparing adult early and late-onset CVI: HVFD spectrum and severity distributions.

We analyzed the data using both graphical and statistical methods to investigate potential differences in HVFD expression between adults with early-onset CVI and late-onset CVI.

## 4. Results

HVFD spectrum and severity distributions were visualized using box-and-whisker plots, which display outliers (dots), trimmed minimum and maximum ranges (whiskers), interquartile range (box edges), median (line inside the box), and mean (✕).

### 4.1. HVFD spectrum and severity distributions for early and late-onset brain injury are significantly distinct from those of adult neurotypicals

**Fig 1:**
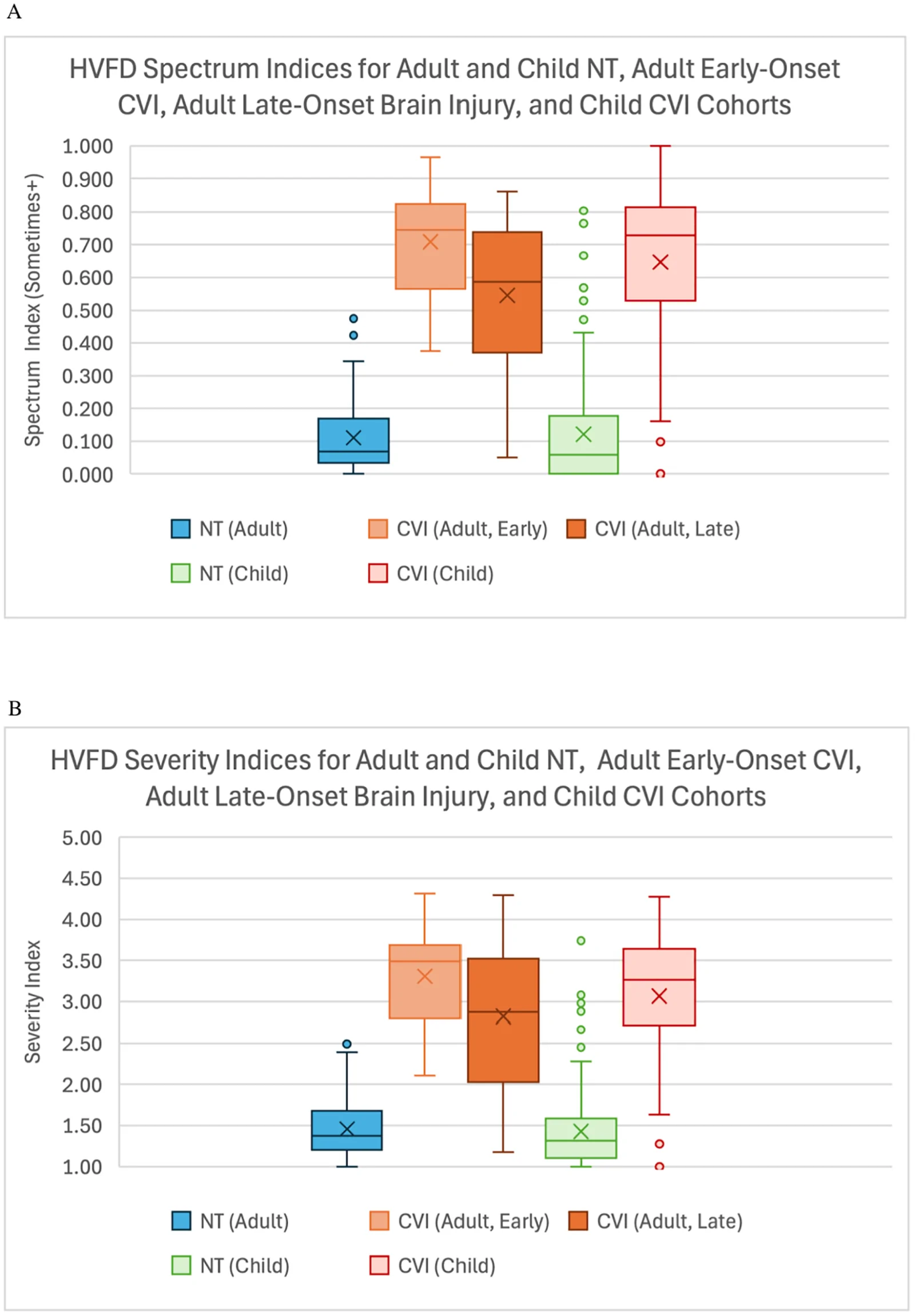
Box-and-whisker plots showing the HVFD. (A) Spectrum Index and (B) Severity Index Distributions for the following cohorts: (1) adult neurotypicals (n = 67); (2) adult early-onset CVI (n = 29); (3) adult late-onset CVI (n = 22); (4) pediatric neurotypicals (n = 120); and (5) pediatric CVI (n = 92). Note: The outliers seen in the NT (Child) and the Child (CVI) cohorts have been explained in Chandna et al., 2024.

Fig 1A shows that adults with early-onset CVI (A–Early Onset) and late-onset brain injury (A–Late Onset) have widely distributed Spectrum Index values, while adult neurotypicals (ANT) are tightly clustered around a low median (0.07). The NT group had only two outliers (0.42 and 0.48), both below the 1st quartile of either CVI group. Median differences are substantial: A–Early Onset vs. ANT (Δ = 0.68), A–Late Onset vs. ANT (Δ = 0.52). These differences are statistically significant: MWU p-values: A–Early Onset vs. ANT <<0.00001 (8.9e-14); A–Late Onset vs. ANT <<0.00001 (5.6e-9).

Fig 1B shows a similar pattern in Severity Index distributions. A–Early Onset severity ranges from 2.1 to 4.3, with the interquartile range between 3.0 and 3.7. ANT severity is concentrated between 1.0 and 2.5 (IQR: 1.4–1.7). Median differences are large and significant: A–Early Onset vs. ANT (Δ = 2.1, p <<0.00001 [3.9e-14]); A–Late Onset vs. ANT (Δ = 1.5, p <<0.00001 [2.7e-8]). No significant difference was observed between the two adult CVI groups.

### 4.2. Adult early-onset CVI spectrum and severity distributions are similar to children with CVI

Spectrum Index distributions for A–Early Onset (n = 29) and child CVI (n = 92) are highly similar, as shown in Fig 1A. The medians and interquartile ranges nearly overlap. The MWU test confirms no significant difference (p = 0.63), suggesting similar HVFD spectrum profiles.

Fig 1B shows a slightly higher Severity Index median for A–Early Onset (3.5) than C–Early Onset (3.3), but this difference is also not significant (p = 0.77). These findings indicate that HVFD expression in adults with early-onset CVI closely resembles that in children with CVI.

**Fig 2:**
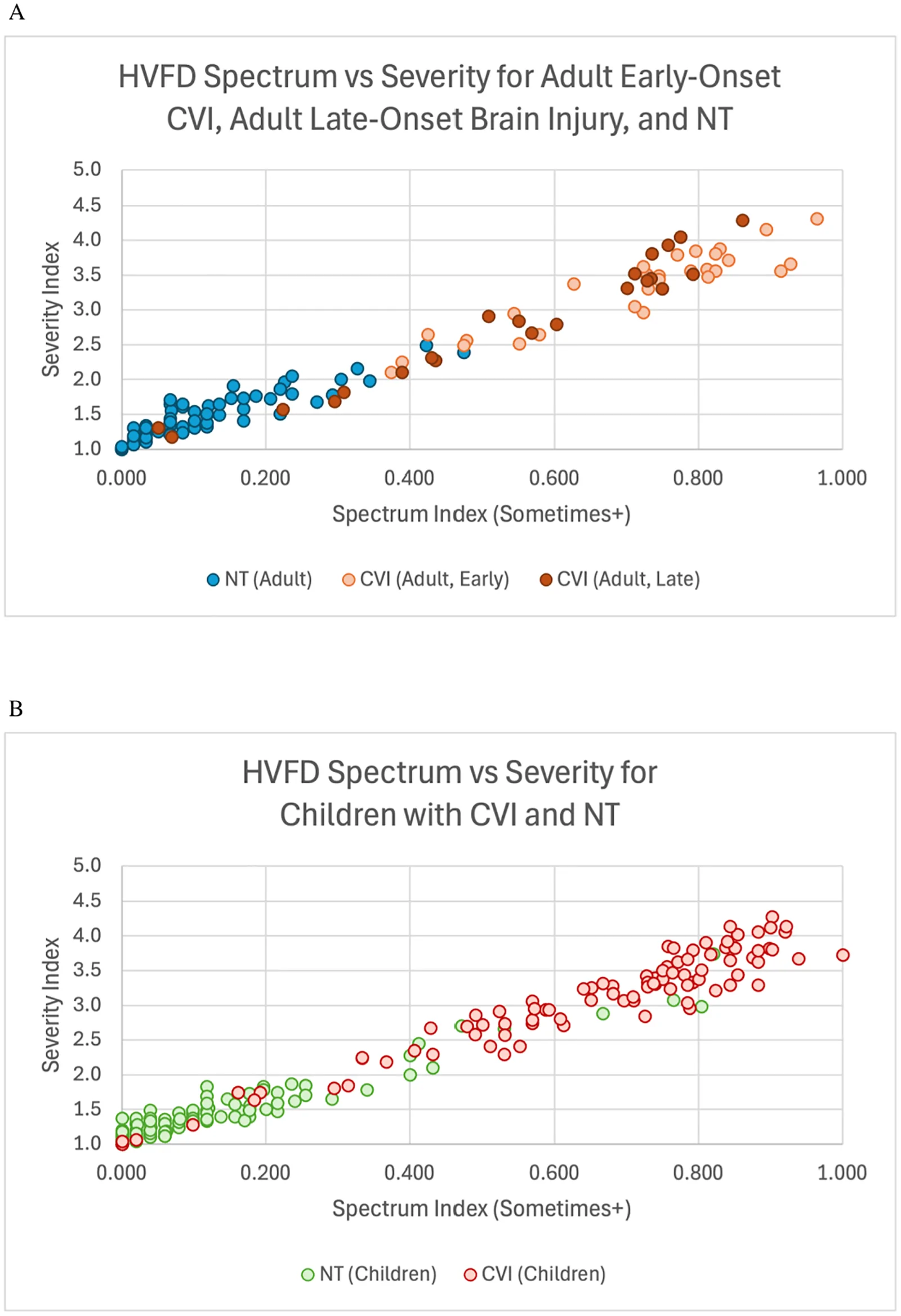
Comparison of spectrum vs. severity scatterplots for: (A) Adult early-onset CVI (n = 29), adult late-onset brain injury (n = 22), and adult neurotypicals (n = 67); and (B) Child CVI and child neurotypicals.

Plotting spectrum versus severity, as seen in Fig 2, provides deep insights into the relationship between these two metrics and provides context for an individual’s HVFD expression profile. As previously reported, the relationship is strongly linear for HVFD expression in children with CVI and neurotypical children [4]. Linear regression for A–Early Onset and ANT (Fig 2A) and C–Early Onset and CNT (Fig 2B) gave high R^2^ values (0.969 and 0.969, respectively), with similar intercepts (1.12 and 1.07) and slopes (3.08 and 3.07). This indicates that CVI symptoms manifest as HVFDs with essentially the same linear relationship for spectrum and severity for both early-onset adults and children. Interestingly, when plotting HVFD severity versus spectrum for adult late-onset brain injuries, the points nestled among a very similar linear regression in close facsimile to both A–Early Onset and C–Early Onset.

### 4.3. HVFD expression among adults with late-onset brain injuries shows distinct but not significant characteristic differences from adults with early-onset CVI

As seen in Fig 1A and 1B, A–Late Onset (n = 22) spectrum and severity distributions appeared visually to be skewed lower than A–Early Onset (n = 29), with a longer lower tail in spectrum, and a broader interquartile range and lower median for severity. The separation of medians between A–Early Onset and A–Late Onset is relatively small (Δ median for spectrum: 0.16, severity: 0.62) compared to A–Early Onset and ANT or A–Late Onset and ANT (see section 4.1). MWU test between A–Late Onset and A–Early Onset shows no significant differences, p-value ≫ 0.00001 (spectrum: 1.5e-2, severity: 6.5e-1).

We included A–Late Onset in the plot of spectrum and severity in Fig 2A for A–Early Onset and ANT, and the results were also strongly collinear and very similar to A–Early Onset and ANT alone (R^2^ = 0.964, slope = 3.16, y-intercept = 1.09). We did not see differences of HVFD expression between A–Late Onset and A–Early Onset or A–Late Onset and C–Early Onset (Fig 2B) as hypothesized at the end of Section 1, regarding diffuse damage (A–Early Onset) versus localized damage (A–Late Onset). For example, there could have been more data points with: (1) low spectrum, high severity deficits as one might expect from late-onset adult brain injuries; or (2) high spectrum, low severity diffuse deficits one might anticipate from A–Early Onset. Furthermore, the linear regression for A–Late Onset, A–Early Onset, and ANT strongly resembles the linear regression for CVI among children and the CNT group.

Among the A–Late Onset cohort, two participants present spectrum and severity metrics consistent with neurotypicals, and three more who are between the 3rd quartile and trimmed maximum spectrum and severity range for adult neurotypicals. We identify four of these participants as outliers and cross-reference Table 4 and discuss our interpretation of these findings in light of the participants’ medical conditions below.

### 4.4. Adult early-onset CVI and adult late-onset brain injury HVFD expression profiles are similar to each other and with child CVI HVFD expression

**Fig 3:**
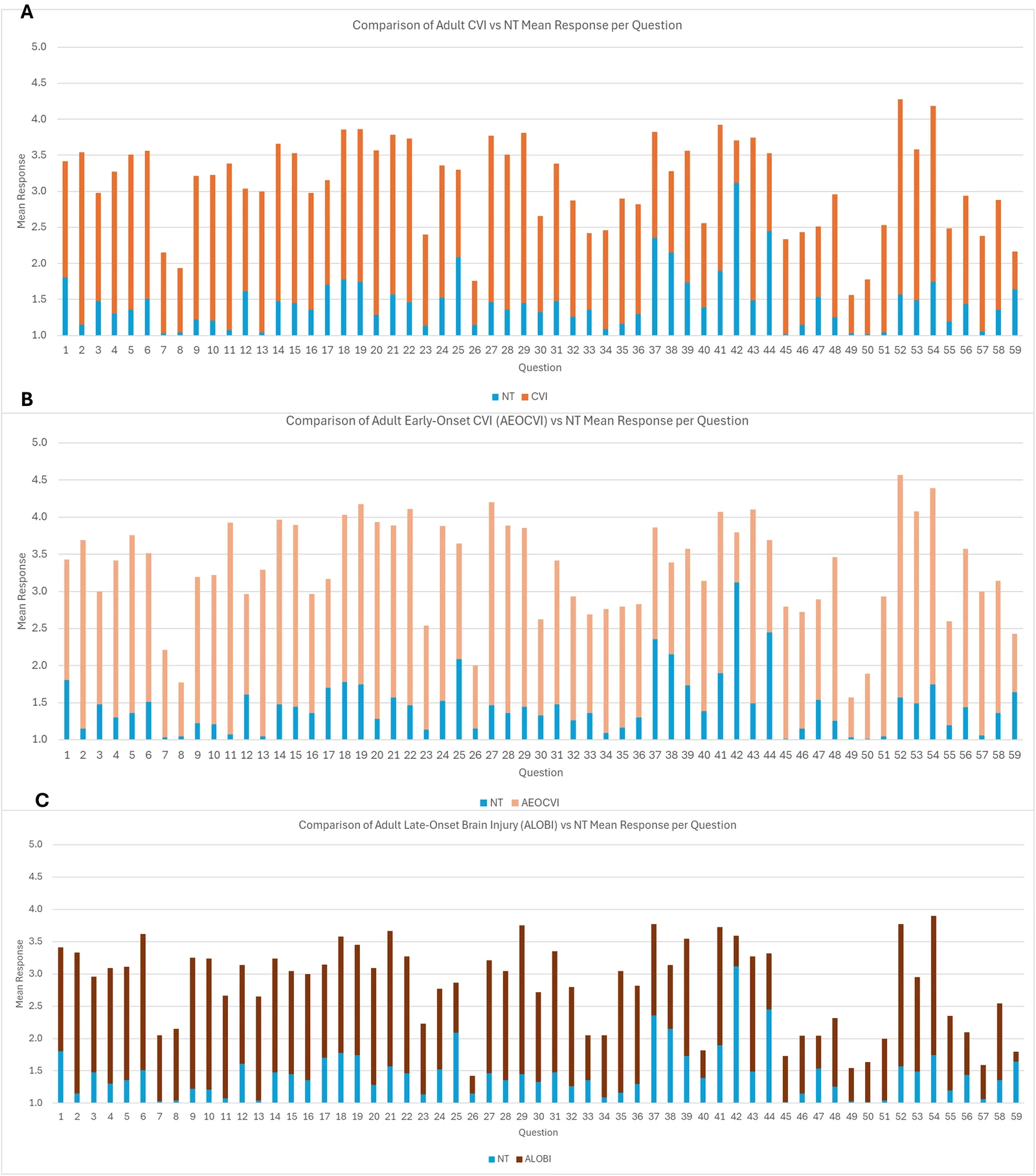
Comparison of mean responses shown in stacked column plots: (A) Adult CVI and adult neurotypicals; (B) Adult early-onset brain injury and adult neurotypicals; and (C) adult late-onset brain injury and adult neurotypicals. There is an underlying emergent pattern in the peaks and troughs of the responses, with deviations from the pattern indicating possible insights into CVI’s effects on different demographics based on etiology.

Column plots of mean responses to the HVFQI instruments show that neurotypical adults (blue columns in Fig 3A and 3B) and children (green columns in Fig 3C) express higher visual functional deficits similarly, with the equivalent peaks and troughs corresponding with small variances. A moderate difference (Δ = 0.9) can be seen with Q26 which in the adult inventory is: *“Do you find copying words or drawings to and from a screen or a document, time consuming and difficult?”* and *“Does your child find copying words or drawings time consuming and difficult?”* in the child instruments. In children (in our cohort 4–18 years old), the mean neurotypical response is approximately 2.1 (likely due to age and language developmental delays seen in children with CVI), whereas the mean neurotypical response in adults is 1.2. A strong difference (Δ = 1.8) occurs with Q42, which states for the adult inventory: *“Do you prefer quiet places or open countryside in comparison to busy cities?”* and in the child inventories: *“Do quiet places/open countryside cause better behaviour?”*

Mean responses from A–Early Onset (light orange columns in Fig 3A) and A–Late Onset (brown columns in Fig 3B) show more variance, likely due to the smaller sample sizes. The following questions are examples of moderate differences (Δ ≥ 1.1) in the responses:

Q11. Have you walked out in front of traffic because you have difficulty judging moving traffic?

Q53. Do you have difficulty identifying objects when they are on a similar background, such as a white T-shirt on a white sheet? (or black shoes on a black background/in a dark cupboard)

Q56. Do you have difficulty recognising an object if it is partially hidden (or not fully visible), or viewed from an unusual angle? (e.g., a bicycle hidden partly behind a wall)

Q57. Do you have difficulty recognising right and left shoes?

For all four questions, A–Late Onset responses are lower than A–Early Onset, however, other questions with similar functional deficits have similar responses, so further investigation is warranted to understand if this difference is significant, and if so, what might be the cause(s).

Mean responses from adult CVIers (light and dark orange columns in Fig 3A and 3B, for A–Early Onset and A–Late Onset, respectively) and parents of child CVIers (pink columns in Fig 3C) are also generally similar, with a few small differences in addition to those previously noted in neurotypical responses. Q41 shows a moderate difference (Δ = 0.9) between children (3.0) and adults (3.9) with CVI and has the following text: (child inventories) *“Do rooms with a lot of clutter cause difficult behaviour?”*, and (adult inventories) *“Do rooms with a lot of clutter cause you difficulty?”*, showing greater sensitivity to cluttered rooms amongst adults.

## 5. Discussion

This prospective controlled study represents a significant step in advancing our understanding of the clinical symptom complex of HVFDs and visual acuity impairment in CVI in adults, marking the first systematic examination of these issues in adults with early-onset CVI. By extending the focus from children to adults, including those with late-onset brain injury, this study breaks new ground in the consequences of brain injury research. Our findings offer compelling evidence of the persistence of visual impairments beyond childhood in CVI, underscoring the long-term nature of these deficits. The comparative analysis of HVFD Spectrum and Severity Indices provides valuable insights into both the shared and distinct features of visual dysfunction across three distinct cohorts: children with CVI, adults with early-onset CVI, and adults with late-onset brain injury. Notably, the study of late-onset brain injury highlights the characterization of HVFDs in this population, underscoring the similarity with HVFDs in CVI and the need for further investigation and sparking important discussions around appropriate terminology. The key findings from this study are as follows. These results further highlight the efficacy of the HVFQI as a valuable clinical tool for detecting CVI-related HVFDs in both children and adults with early-onset CVI. When used in conjunction with the individual’s context and specific circumstances as an adult, the indices provide a personalized picture that is crucial for accurate characterization at diagnosis, follow-up, and targeted habilitation therapy.

### 5.1. Comparison of adults with early-onset CVI and neurotypical adults using the HVFQI-59Q

The new rephrased HVFQI-59Q reliably elicits HVFDs in adults with early onset CVI, clearly distinguishing Adult early-onset CVIers from adult Neurotypicals. The 2-dimensional view of the effect of HVFDs with Spectrum and Severity Indices revealed significant differences between adults with early-onset CVI and neurotypical adults. Adults with early-onset CVI exhibited broader distributions and higher median values in both indices compared to neurotypical adults, who had tightly clustered low values. These differences underscore the effectiveness of the indices in distinguishing between cohorts and suggest that CVI, regardless of onset, imposes a measurable and widespread impact on visual functions in adults. Though the need for early diagnosis has been emphasized with guidelines [19,20,81,82] and criteria [8], delays in diagnosis are common in CVI. At Least 3% of children in primary school were undiagnosed for CVI despite having at least one CVI related HVFD problem [83], one case study described diagnosis delayed until age 11 years [84] and a review highlighed the impact of underdiagnosis of CVI in children with CVI [37]. Such delays in diagnosis extend to late adolescent years and adulthood. The need to recognize that children with CVI grow into adults with persistent CVI has been emphasized in a recent editorial [2]. CVI may also manifest for the first time in adults and has been reported for genetic conditions [22]. The HVFQI-59Q has the potential for use as a diagnostic tool for HVFDs in adults with early-onset CVI highlighting the importance of clinical CVI screening and potential diagnosis, training, and treatment for these individuals. Further work with a larger cohort and controlled studies will clarify its role and elicit the most discriminatory questions as a screener for implementation in clinical practice.

### 5.2. Similarity between early-onset CVI in adults and children

The study found striking similarities in HVFD profiles between adults with early-onset CVI and children with CVI, indicating the persistence of HVFDs through life, confirming our hypothesis that children grow into adults with HVFDS, albeit they may show potential differences in spectrum and severity with time. In our study, both groups displayed comparable distributions in spectrum and severity indices, suggesting that the nature of visual deficits may remain consistent over time. These comparisons were based on harmonised indices derived from the shared 51-item core across adult and paediatric inventories and should be interpreted in that context. This consistency supports the idea that the indices capture core characteristics of CVI across age groups. Although minor differences were noted, such as slightly higher severity among early-onset CVI in adults, these were not statistically significant but deserve further study. The trend towards higher severity could be due to the relatively more complex visual environment in adult life where adjustments that may have been made by families and educators, TVIs for children within CVI may not be available as adults with CVI. Our study included adults with early-onset CVI diagnosed in childhood. We know that CVI in adults may be suspected for the first time in adults with specific genetic syndromes destined to manifest in later life [2,22,85] and to overcome this limitation we will recruit such adult participants.

### 5.3. Comparison of adults with late-onset brain injury and neurotypical adults using the HVFQI-59Q

We have characterized HVFDs for the first time in adults based on a prospective controlled study and have detected a statistically significant difference in HVFD expression between adults with late-onset brain injuries and neurotypical adults. This provides evidence that (1) adults with late-onset brain injuries express higher visual functional deficits; and (2) that spectrum and severity characterizations of those deficits in A–Late Onset are distinct from HVFDs in ANT in a similar fashion as A–Early Onset are from ANT cohorts. The HVFQI-59Q shows significant potential as a diagnostic tool for identifying HVFDs, also in adults with late-onset brain injury. Further research involving larger cohorts and controlled studies will refine its role and help identify the most effective questions for use as a screening tool in clinical practice.

The 4 outliers within the spectrum and severity of A-Late Onset (see Table 4) are included in the study for transparency as we probe the possible reasons; 2 are between the median and 3rd quartile, and 2 more are between the 3rd quartile and trimmed maximum. There is a 5th data point on the very cusp of the trimmed maximum, which we do not include in this discussion for outliers. The outliers indicate the following possibilities. The site of primary neurological insult did not involve higher visual functions and thus showed no deficits; there may have been recovery or effective strategies developed to “mask” the HVFDs or HVFQI-59Q needs reassessment. The primary neurological diagnosis may exclude HVFDs for participants 1, 3, and 15, but not necessarily for participant nine. The interval between the brain injury event and the data collection was very variable even for a similar primary neurological diagnosis: eight years (#1); one year (#3); nine years (#9) and six years (#15); and recovery from HVFDs would also depend on several additional factors (such as age, treatment, rehabilitation) and this reason is likely to remain unproven. Effective strategies or accommodations may mask HVFDs [86] and more so in CVI, as CVIers may be unaware of their HVFDs. Similar to the A–Late Onset cohort, 6 outliers within the child NTs were reported [3,4]. Outliers, commonly reported within questionnaire studies, are to be expected, and analytical methods have been suggested [87,88]. Further work with a larger cohort will help in reviewing outliers and determining whether the HVFQI-59 needs modification. However, in our present work the evidence for the presence of HVFDs, provided for the first time in individuals with late-onset brain injuries, strongly indicates that these injuries may lead to visual deficits similar to those seen in CVI, underscoring the critical need for clinical CVI screening, diagnosis, and targeted treatment for these individuals. It also calls for an informed discussion on terminology for adults with late-onset brain injury with HVFDs and vision impairment.

### 5.4. Patterns in late-onset brain injury

Adults with late-onset brain injuries (A–Late Onset) exhibited HVFD profiles that were visually distinct from those with early-onset CVI (Fig 1A and 1B), though the differences were not statistically significant. The A–Late Onset cohort appeared visually to show broader range of severity and lower median values, suggesting a trend, but not enough to indicate fundamentally different patterns of visual deficits, likely due to sample size limitations. This finding implies that late-onset brain injuries may result in visual deficits that closely resemble those caused by CVI, underscoring the need for these participants to seek clinical CVI screening and potentially diagnosis, training, and treatment. The results also indicate that HVFDs are not localized and, in our cohort, have a broader range. This does not, as yet, entirely agree with our hypotheses that A–Late Onset’s with localised brain injury, such as tumors and infarcts (stroke), will manifest a narrower spectrum of HVFDs whereas the opposite may be true for traumatic brain injury. This may well be proven with a larger cohort with the ability to look at specific etiologies. However, HVFDs are best described as visually guided behaviors mediated through activation of several areas of the brain and it is likely that this is true representation of HVFDs and concur with the concept of multiple areas of the brain involved in visually guided behaviors where synchronous neuronal activity occurs over short timescales within and between cortical areas and has been observed as a correlate of perception [89] and, established processes within the brain allow different brain regions to stay connected and communicate efficiently, ensuring coordinated brain activity despite variations in signal travel times “similar to the backplane of a mainframe computer” [90]. Differential activation of dorsal and ventral pathways in CVI during a visual search task in CVI participants has been reported, though more work is needed to establish the reason for this effect [91].

### 5.5. Common trends across ages and conditions

A spectrum of HVFDs has been described previously in children with CVI [14,20,92], and [93] described the spectrum and severity of the overall condition but not of HVFDs. The addition of quantitative spectrum *and* severity indices for HVFDs is a unique feature of analysis resulting from the use of HVFQI instruments in children and now in adults where we report the two indices and their relationship to provide a two-dimensional view of CVI across the ages (4–70 years) for CVI and also for late-onset brain injury in adults. The relationship between spectrum and severity appeared to follow a similar linear trend across all groups, regardless of age. This consistency indicates a shared underlying mechanism driving visual deficits for children and adults with early-onset CVI. Remarkably, the collinearities between severity and spectrum were nearly identical when comparing adult early-onset CVI, late-onset brain injury, and neurotypical cohorts, and when comparing child early-onset CVI and child neurotypical cohorts. This indicates that HVFD expression among A–Late Onset behaves as CVI-related HVFDs of all age categories. Again, we emphasize the need to sharpen our terminology to cover all aspects of the visual consequences of brain injury, both early and late onset.

### 5.6. Potential for targeted question refinement

Generally, responses between ANTs and Child NTs were similar, as were responses between A–Early Onset and C–Early Onset. Differences in mean responses to specific inventory questions, potentially influenced by age-related or cultural factors, raised awareness of questions that could be rephrased for improvement. One question had notable differences in responses among neurotypicals, where neurotypical adults prefer quiet places as opposed to noisy areas, whereas more parents reported no noticeable improvement of behavior for neurotypical children in quiet areas. A few more questions showed notably different responses among different CVI age groups, for example, the higher severity responses for adults with CVI on the impact of clutter may reflect societal influences as the adult cohort was mostly from Western, English-speaking countries where tidying up is seen as a hallmark of a responsible adult. These expectations would raise the pressure for CVIers to feel more anxiety and distinction from their neurotypical peers, whereas children are subject to lower expectations. These questions may need to be rephrased in the future and retested. Such insights suggest opportunities for refining the HVFQI instrument to account for developmental and contextual variations. Importantly, at the detail level of individual questions, responses from A–Late Onset closely matched A–Early Onset responses, adding more evidence that the A–Late Onset cohort presents HVFDs at distinctly higher levels than ANT, and the HVFD profiles are similar to CVI-related HVFD profiles.

### 5.7. Limitations

This study had several limitations. The relatively small sample sizes, particularly in the A–Late Onset and A–Early Onset groups, may limit generalizability and the detection of subtle group differences; however, as the first prospective controlled study of its kind, it provides an important foundation for future, larger, and longitudinal research [94]. While CVI has multiple causes, the participants in this study represent a broad and typical CVI cohort. The HVFQI is a semi-structured questionnaire-based instrument, and such tools are widely used across all branches of medicine for clinical assessment [95]. In CVI, questionnaire inventories have been extensively applied and validated supporting their relevance in capturing HVFDs and remain the most reliable method in the absence of validated clinical objective assessment tools for HVFDs in CVI [96]. Where relevant, carers supported responses to reduce bias from limited self-awareness, a validated method in populations with communication challenges [97]. Variability in time since injury among adult participants may have influenced HVFD expression, but this heterogeneity reflects real-world clinical populations [98]. Finally, although objective clinical assessments were not included, the strong differentiation across cohorts supports the HVFQI’s value as a clinical screening tool.

We acknowledge that the design of our study introduces the potential of interviewer or expectation bias, particularly given the subjective nature of symptom reporting. However, there is no evidence that this systematically affected the results: responses span the expected spectrum, with some degree of overlap across groups, and no absolute separation between participants with CVI experiencing maximal symptoms and those with milder challenges. Statistical patterns also show no systematic bias, and our procedures and recruitment criteria are consistent with several other questionnaire- and inventory-based studies where blinding was not implemented, which has been discussed in the manuscript.

Potential confounding by demographic and clinical differences between adult groups should also be considered. In particular, age and visual acuity may influence self-reported functional outcomes. Years of education were not systematically collected and could therefore not be examined. Because this was an exploratory study with modest subgroup sizes and incomplete covariate information, the primary analyses were based on pre-specified non-parametric comparisons of derived HVFD indices rather than covariate-adjusted modelling. These factors should therefore be borne in mind when interpreting between-group comparisons.

A further limitation concerns comparison with the historic paediatric cohorts. Adult and paediatric data were compared after harmonisation onto the shared 51-item core, rather than by direct comparison of the full adult and paediatric questionnaires. Although this preserved continuity of the principal behavioural constructs, minor differences in wording, response framing, and developmental context remained, and some information from later-added adult items was necessarily excluded. Adult–child comparisons should therefore be interpreted as comparisons of harmonised indices across a common core item set rather than strict equivalence between identical instruments.

The psychometric findings presented here provide encouraging preliminary support for the adult HVFQI-59Q, including strong internal consistency and good discrimination between adult neurologically impaired and neurotypical groups. However, this should not be regarded as a full validation study of the adult instrument. Further work in larger adult cohorts, including formal assessment of test–retest reliability, domain structure, and clinical utility across different settings, is required.

## 6. Conclusion: Implications for clinical understanding and future research

We have presented unique findings from populations of adults with visual consequences of brain injury that have not been studied and reported before, contributing to and extending the knowledge in this field. The findings from this study confirm that CVI leads to broad and severe HVFDs that are clearly distinguishable from neurotypical patterns among early-onset CVI in adults in a similar fashion to that of early-onset CVI in children and in adults with late-onset brain injury. Additionally, the consistent relationship between spectrum and severity across age groups indicates that these metrics reliably capture the extent and nature of CVI-related deficits. This consistency also suggests that HVFD profiles in A–Late Onset individuals are likely related to CVI, highlighting the need for a more precise terminology, as current terms do not fully describe the manifestations observed in this study. While the study identified some potential differences between groups (*e.g.,* HVFD profiles for early-onset CVI and HVFD profiles from late-onset brain injury), larger sample sizes are needed to confirm these observations and further characterize the long-term impact of CVI on functional vision. Understanding these nuances can increase our understanding, guide diagnosis; more tailored interventions and support strategies for individuals across different life stages.

## Supporting information

S1 Appendix Examples of questions rephrased for adults from the paediatric HVFQI, and a structured table documenting the principal paediatric-to-adult adaptations of the HVFQI-59Q. (DOCX)

S1 Checklist PLOS questionnaire on inclusivity in global research, completed for this study. (DOCX)
