# Supplementary material for: Persistence of higher visual function deficits in adults with early-onset cerebral visual impairment: A comparative study
Source: PLoS One. 2026 Aug 21;21(8):e0354174. doi: 10.1371/journal.pone.0354174 (PMC13497256; doi:10.1371/journal.pone.0354174)
Supplement: Supplementary file 1 [file pone.0354174.s001.docx]

# **S1 Appendix**

Examples of Questions rephrased for adults from HVFQI for children aged 4-18 years (Chandna et al 2012; Chandna et al 2024).

Original Question followed by rephrased Questions for different visual domains.

1-Spatial Perception and Lower Vision Field

Does your child have difficulty walking downstairs?

Do you have difficulty walking downstairs for visual reasons?

2- Spatial Perception and Reading

Does your child have difficulty finding the beginning of a line when reading?

Do you have difficulty finding the beginning of a line when reading a printed book?

3- Spatial Perception and Movement

Does your child have difficulty catching a ball ?

Do you have difficulty catching something that is thrown to you? (*e.g.* a ball)

4- Visually Directed Reaching ( Visuomotor integration)

Does your child grasp objects incorrectly when picking them up?

When picking up an object, do you grasp it incorrectly? (*e.g.* do you miss or knock the object over?)

**Adaptation of the paediatric HVFQI to the adult HVFQI-59Q**

| **Child HVFQI-58Q item(s)** | **Adult HVFQI-59Q item(s)** | **Nature of adaptation** | **Rationale (adult-specific demands)** |
| --- | --- | --- | --- |
| Q1–Q15 | Q1–Q15 | Minor wording updates only; constructs unchanged. | Converted to adult self-report phrasing; retained age-general constructs (mobility/lower VF, reading, motion, visual search). |
| Q4: Do you trip at the edges of pavements going down? (stepping off the bus) | Q4: Do you trip at the edges of pavements going down or have difficulty stepping off buses or other public transport? | Example broadened. | Extends from child-centric ‘bus’ example to adult public transport contexts. |
| Q11: …difficulty seeing the cars moving towards you | Q11: …difficulty judging moving traffic | Functional wording broadened. | Captures adult task of traffic judgement in independent mobility, not just detecting approaching cars. |
| Q13: Do you have difficulty seeing the two sides of an open book while reading? | Q13: Do you have difficulty seeing the two sides of an open book or filling out two sides of a form? | Adult task added. | Adds adult document completion demands while preserving the underlying ‘two-page/dual-column’ visual span construct. |
| Q14: …seeing detail… when inside of a moving car (as a passenger) | Q14: …when riding as a passenger in a moving vehicle | Travel context generalised. | Broadens beyond ‘car’ to typical adult travel contexts. |
| Q16: Do you enjoy watching fast moving TV and film images? (or prefer to watch slow moving TV and film images?) | Q16: Do you avoid watching fast moving TV and film images? AND Q17: Do you prefer to watch slow moving TV and film images? | One item split into two (adds one scored item). | Separates avoidance of fast-moving content from preference for slow-moving content for clearer adult self-report. |
| Q17: Do you have difficulty catching a ball? | Q18: Do you have difficulty catching something that is thrown to you? (e.g. a ball) | Example broadened. | Generalises from child play to a wider adult catching task. |
| Q22: …places that are well known… (e.g. at school, in your neighborhood) | Q23: …places well known… (e.g. neighbourhood; workplace; transit hubs/stops) | Adult-specific examples added. | Reflects adult independence, employment, and travel routines. |
| Q25: Do you like sitting close to the television… because you find it easier to watch or understand? | Q26: Do you sit close to the television? (e.g. less than about 30 cm) | Preference to behaviour wording. | Frames as a behavioural report rather than preference, improving adult interpretability. |
| Q26: …copying words or drawings from the board or screen… | Q27: …copying words or drawings to and from a screen or a document… | School-based task to adult screen/document task. | Replaces ‘board’ wording with workplace and administrative screen–document copying demands. |
| Q28: Do you find uneven ground difficult to walk over? | Q29: …uneven ground… (e.g. grass, hiking trail) | Adult example added. | Extends to typical adult outdoor mobility scenarios. |
| Q36: …keep to a task if there are distractions… | Q37: …keep to a challenging task if there are distractions… | Task demand specified. | Aligns with higher attentional demands typical of adult work and independent living. |
| Q42: difficulty getting around in a supermarket/busy shop? | Q43: difficulty getting around in a busy place (e.g. supermarket, busy street, airport) | Environment broadened. | Includes adult navigation in travel and crowded public environments. |
| Q47: …happy/annoyed/sad by looking at their face? | Q48: difficulty understanding the meaning of facial expressions (e.g. smiling/annoyed/sad) | Wording clarified. | Maintains the social interpretation construct with clearer adult phrasing. |
| Q51: difficulty finding out where a sound is coming from? | Q59: difficulty identifying where sound is coming from? | Item reordered; minor wording change. | Construct retained; moved to the end of the adult inventory after the higher-order recognition items (Q52–Q58). |
| Q52–Q58 | Q52–Q58 | Minor wording updates only; constructs unchanged. | These recognition, crowding and generalisation items are broadly age-general; adult version mainly polishes wording/examples. |
| Side-indicator sub-items (e.g. Q7.1, Q8.1, Q12.1, Q13.1) | Corresponding adult side-indicator sub-items | Response options expanded in some items (e.g. adding ‘both’). | Supports clearer reporting where difficulty may be bilateral/non-lateralised. |
